# Detection and quantitation of ferritinophagy using HaloTag tracing

**DOI:** 10.1016/j.jbc.2025.111016

**Published:** 2025-12-06

**Authors:** Sachin K. Kempelingaiah, Alexandra J. Straus, Grace Mavodza, Can E. Senkal

**Affiliations:** 1 Department of Cellular, Molecular, and Genetic Medicine, Virginia Commonwealth University School of Medicine, Richmond, Virginia, USA; 2 C. Kenneth and Dianne Wright Center for Clinical and Translational Research, Virginia Commonwealth University School of Medicine, Richmond, Virginia, USA; 3 Massey Comprehensive Cancer Center, Virginia Commonwealth University School of Medicine, Richmond, Virginia, USA

**Keywords:** ferritinophagy, FTH1, ferritin, HaloTag, NCOA4

## Abstract

Iron is an essential element required for critical processes, such as oxygen transport, energy generation, and DNA synthesis. To be incorporated as a cofactor, iron that is stored in the cytosol within ferritin needs to be liberated by ferritinophagy. Ferritinophagy is an autophagic process in which ferritin is targeted to the lysosomes, through its interaction with nuclear receptor coactivator 4 for degradation and release of labile iron. Despite its involvement in neurodegenerative diseases, anemia, cancer, and insulin resistance, a specific and sensitive method to detect ferritinophagy has been lacking. To detect and quantitate ferritinophagic flux, we generated a Halo-tagged ferritin heavy chain 1 (FTH1) construct and took advantage of stabilization of Halo fragment in the presence of its fluorescently labeled ligand. Stably expressed Halo-FTH1 operated identical to its endogenous counterpart. More importantly, using pulse-chase settings, lysosomal accumulation of Halo fragment after induction of ferritinophagy was detected and quantitated by in-gel fluorescence, immunoblotting, and microscopic analyses. Finally, we found that silencing of nuclear receptor coactivator 4 prevented accumulation of tetramethylrhodamine-Halo fragment and degradation of endogenous FTH1 under ferritinophagic conditions, confirming the specificity of our assay. Together, the HaloTag-FTH1 tool we generated can be used to specifically detect and quantitate ferritinophagy in mammalian cells with a fluorescent Halo ligand, and this approach can be instrumental in studies focusing on cellular iron metabolism.

Autophagy is a lysosome-based degradation system in eukaryotes that is essential for cellular homeostasis as part of the intracellular quality control and remodeling during environmental adaptation (1). Macroautophagy is characterized by the formation of double membrane vesicles, called autophagosomes, which sequester long-lived proteins, damaged organelles, or invasive microbes within the cytosol. Autophagosomes fuse with lysosomes, which degrade sequestered products and release the digested material into the cytosol (2). Autophagic processes recycle the macromolecular constituents to generate energy, to maintain cell viability under unfavorable conditions, to protect the cell during various conditions of stress (1) or regulate cell death (3). In addition to macroautophagy, cells can degrade specific cargo in the lysosomes through receptors and adaptors (4). Ferritinophagy is a specialized autophagic process necessary for the maintenance of iron homeostasis in cells (5). In ferritinophagy, ferritin is targeted to the lysosomes for degradation and the generation of labile iron.

Ferritin is a highly conserved iron storage protein complex that possesses the ability to store up to 4500 Fe^3+^ atoms within its inorganic mineral core (6, 7). Ferritin is a 24-subunit cage-like complex composed of ferritin heavy chain 1 (FTH1) and ferritin light chain (FTL) subunits (8, 9). Ferritin levels are tightly regulated at the post-transcriptional level by iron regulation proteins (IRPs) 1 and 2 (10). This type of regulation is crucial to cellular fitness as high levels of labile iron pool within the cytosol can undergo the Fenton reaction, where reactive oxygen species react with reactive ferrous iron mediating phospholipid peroxidation and triggering ferroptosis, a form of programmed cell death (11). Therefore, labile iron pool generation through ferritinophagy is crucial for ferroptosis as described previously (12, 13).

Nuclear receptor coactivator 4 (NCOA4) has been identified as the autophagy receptor for ferritin, where NCOA4 binds to FTH1 subunit and facilitates lysosomal targeting and degradation of ferritin, thereby releasing stored ferrous iron into the cytoplasm (14). Hence, NCOA4 is one of the key proteins for ferritinophagy as knockdown of NCOA4 prevents ferritinophagy and ferroptosis in several cellular models (13, 14, 15). NCOA4 stability can be regulated by HECT domain– and RLD domain–containing E3 ubiquitin protein ligase 2 (HERC2). Post-translationally, under iron replete conditions, HERC2 directs the degradation of NCOA4 through the ubiquitin protease system, promoting the upregulation of ferritin and sequestration of excess iron in the cytoplasm (16). In iron deplete conditions, HERC2 ubiquitinates F-box and leucine-rich repeat protein 5 (FBXL5), which upregulates the expression of IRPs, promoting ferritinophagy (17). In addition, cellular oxygen levels play a role in mediating the expression of the NCOA4, acting cooperatively with FBXL5 and IRPs (18).

Neurodegenerative diseases, such as Alzheimer’s disease, Huntington’s disease, and Parkinson’s disease are associated with dysregulation of iron homeostasis (19, 20). In addition, dysregulation of ferritinophagy also plays roles in anemia, cancer, and insulin resistance (21, 22). This highlights the importance of understanding NCOA4–FTH1 axis of ferritinophagy in disease. Hence, quantitating ferritinophagic flux is critical in uncovering the underlying mechanisms regulating ferritinophagy (23). However, quantitating autophagic flux in mammalian cells comes with its many limitations. Autophagic flux has widely been monitored *via* fluorescent reporters like red fluorescent protein (RFP)-GFP-LC3 (24) or GFP-RFP-LC3 (25). These reporters can be visualized *via* immunofluorescence microscopy where autophagic flux can be observed by the intensity of the lysosome-resistant RFP *versus* GFP, where GFP is quenched within the lysosomes. However, the success rate and repeatability of these assays depends on altering the luminal pH of lysosomes with nonsaturating concentrations of lysosomotropic agents (26), and some require the use of a lysosomal inhibitor control, which can limit the assay’s dynamic range (27). Moreover, the total amount of induced degradation is often relatively finite (28). In most cases, a proper representation of lysosomal degradation *versus* time was proven futile, and failing to address the many issues that come with these assays can lead to misinterpretations of autophagic flux (29).

To overcome these limitations, a HaloTag (Halo)-based reporter processing assay was introduced recently to quantitate autophagic flux. In this assay, stabilization of Halo-tagged LC3 in the presence of exogenously added chloroalkane group, generally a fluorescent HaloTag ligand, was used (29, 30). The stabilized HaloTag–Halo ligand complex accumulated within the lysosome and could be quantified *via* in-gel fluorescence, immunofluorescence microscopy, and immunoblotting.

In this work, we sought to establish a HaloTag-based assay to quantitate ferritinophagic flux. We established cells that stably express N-terminal Halo-tagged FTH1. Halo-FTH1 acted identical to the endogenous FTH1 with respect to its localization and processing in response to ferritinophagy inducers, such as iron chelator deferoxamine (DFO). We demonstrate that tetramethylrhodamine (TMR)-Halo ligand–induced stabilization of Halo-FTH1 lysosomal cleaved product can be quantitated using in-gel fluorescence, immunoblotting, and confocal microscopy. Importantly, genetic silencing of NCOA4 prevented lysosomal degradation of Halo-FTH1 in response to ferritinophagy inducers. Taken together, we establish that Halo-FTH1 expression together with fluorescent anti-Halo ligands can be used to quantitate ferritinophagy.

## Results and discussion

**Figure 1:**
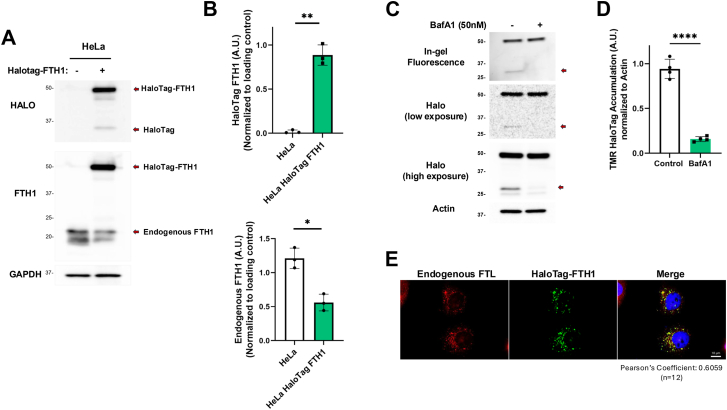
**Generation of Halo-tagged FTH1–expressing cells.***A* and *B*, stable expression of Halo-FTH1 was confirmed. HeLa cells were transduced with lentiviral particles encoding Halo-FTH1, and immunoblotting was performed after selection of stable Halo-FTH1–expressing cells. Band intensities for Halo-FTH1 and endogenous FTH1 were quantitated from three independent experiments. Data represent mean ± SD. Statistical analysis was done by unpaired parametric *t* test ∗∗*p* < 0.001, ∗*p* < 0.05. *C* and *D*, cells stably expressing Halo-FTH1 were treated with 50 nM bafilomycin A1 (BafA1), and Halo-TMR band intensities were quantitated from three independent experiments. Data represent mean ± SD. Statistical analysis was done by unpaired parametric *t* test ∗∗∗∗*p* < 0.001. *E*, Halo-FTH1 and endogenous FTL were visualized using immunofluorescence microscopy (the scale bar represents 10 μm). Nucleus was visualized by DAPI staining. n = 12 in three independent experiments. Pearson’s colocalization coefficient was quantitated using ImageJ. DAPI, 4',6-diamidino-2-phenylindole; FTH1, ferritin heavy chain 1; FTL, ferritin light chain.

Detection of autophagic flux using Halo-LC3 and Halo-KDEL takes advantage of the stability of the HaloTag in the presence of its ligand after autophagolysosomes are formed (31, 32, 33). Ferritinophagy is degradation of ferritin complex in the lysosomes through the autophagy adaptor protein NCOA4 (14, 34). We sought to generate a method to detect ferritinophagic flux using a HaloTag approach. First, we generated HeLa cells that stably express N-terminal Halo-tagged FTH1 and confirmed its expression using anti-Halo and anti-FTH1 antibodies (Fig. 1, *A* and *B*). Of note, endogenous FTH1 protein levels were decreased in Halo-FTH1 expressors compared with the control (Fig. 1*B*, *bottom graph*), suggesting that endogenous FTH1 might be regulated through a protein-sensing mechanism. Rapidly growing cells depend on replenishment of cytosolic iron through ferritinophagy at a basal level to supply iron as a cofactor for iron–sulfur-containing proteins (22). Consistent with this notion, we could detect the cleaved Halo fragment from Halo-FTH1 in basal conditions (Fig. 1*A*). Importantly, inhibition of lysosomal activity with bafilomycin A1 prevented formation of this cleaved product (Fig. 1, *C* and *D*), suggesting the presence of basal ferritinophagy that can be detected by the Halo-FTH1 construct. Next, we identified the cellular localization of Halo-FTH1 using microscopy. Our data show that Halo-FTH1 formed cytosolic puncta that colocalized with the endogenous FTL, detected by anti-FTL antibody (Pearson’s coefficient of 0.6059) (Fig. 1*E*), suggesting that Halo-FTH1 is functional and localizes to the same compartments as the endogenous FTH1.

**Figure 2:**
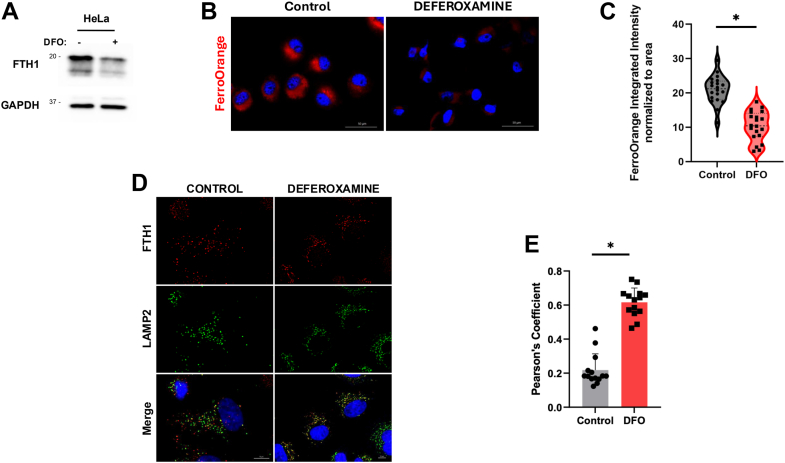
**Deferoxamine (DFO) induced ferritinophagy.***A*, ferritin degradation upon DFO-induced ferritinophagy was detected using immunoblotting. *B* and *C*, labile iron levels were measured by FerroOrange staining and quantitation of average fluorescence as described in *Experimental procedures* section. n = 20 in three independent experiments. Data represent mean ± SD. Statistical analysis was done by unpaired parametric *t* test ∗*p* < 0.0001. *D* and *E*, colocalization of FTH1 and lysosomes (LAMP2) was detected using immunofluorescence microscopy, and Pearson’s colocalization coefficient was quantitated. The scale bar represents 10 μm. n = 14 in three independent experiments. Data represent mean ± SD. Statistical analysis was done by unpaired parametric *t* test ∗*p* < 0.0001. FTH1, ferritin heavy chain 1; LAMP2, lysosome-associated membrane protein 2.

**Figure 3:**
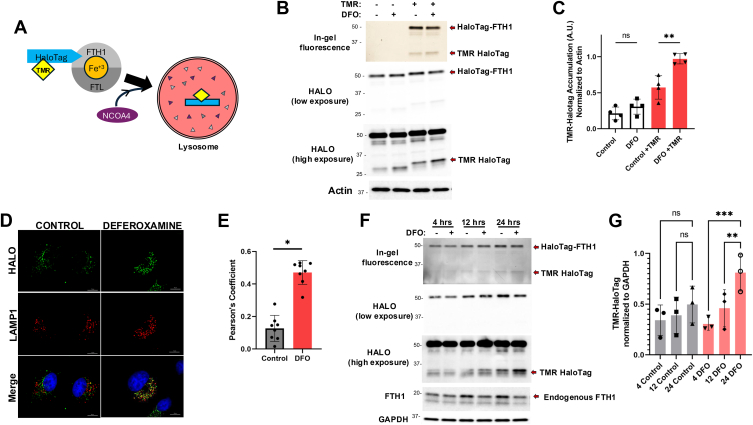
**Detection of ferritinophagy using Halo-FTH1.***A*, schematic representation of Halo-FTH1 processing in ferritinophagy. *B* and *C*, cells stably expressing Halo-FTH1 were treated with deferoxamine (DFO) for 16 h. TMR-labeled Halo ligand (100 nM) was added to the culture media for 2 h. Halo-FTH1 and TMR-Halo complex was detected using in-gel fluorescence and immunoblotting as described in the Experimental procedures section. TMR-HaloTag band intensity was quantitated from three independent experiments. *D* and *E*, colocalization of Halo fragment and lysosomes (LAMP1) was visualized using anti-Halo and anti-LAMP1 antibodies in confocal fluorescence microscope, and Pearson’s colocalization coefficient was quantitated. The scale bar represents 10 μm. n = 8 in three independent experiments. Data represent mean ± SD. Statistical analysis was done by unpaired parametric *t* test ∗*p* < 0.0001. *F* and *G*, cells were treated with DFO for indicated times, and ferritinophagic flux was quantitated by measuring band intensity of Halo fragment. n = 3 independent experiments. Data represent mean ± SD. Statistical analysis was done by two-way ANOVA with Tukey’s multiple comparison tests ns (nonsignificant) *p* > 0.05, ∗∗*p* < 0.05, ∗∗∗*p* < 0.01. FTH1, ferritin heavy chain 1; LAMP2, lysosome-associated membrane protein 1; TMR, tetramethylrhodamine.

Chelation of cytosolic iron using DFO is commonly used to induce ferritinophagy (35, 36). DFO treatment caused degradation of endogenous FTH1 protein (Fig. 2*A*) and a significant decrease in cytosolic iron levels, detected by FerroOrange staining, in HeLa cells (Fig. 2, *B* and *C*). In addition, DFO induced lysosomal localization of endogenous FTH1 (Fig. 2, *D* and *E*), consistent with previous reports. Of note, we observed FTH1 puncta that do not colocalize with lysosomes in basal, unchallenged, conditions. This pool of FTH1 corresponds to the endogenous ferritin complex that stores excess iron as previously described (37). These results suggest that DFO can be used to induce ferritinophagy to test the functional relevance of Halo-FTH1 in this cell line. Next, we used Halo-FTH1–expressing cells, pretreated them with TMR-Halo ligand to stabilize the cleaved Halo fragment in the lysosomes (Fig. 3*A*), similar to previously described Halo-LC3 (29, 30). Upon DFO treatment, we observed a decrease in the full-length Halo-FTH1, accompanied by an increase in the cleaved Halo fragment detected by both in-gel fluorescence and immunoblotting (TMR-HaloTag) (Fig. 3, *B* and *C*). Important to highlight that in-gel fluorescence can only detect fluorescence once TMR ligand is included. In addition, accumulation of stabilized (cleaved) Halo is an accurate representation of the amount of ferritin that is being degraded in the lysosomes, which can be detected at ∼33 kDa in both in-gel fluorescence and immunoblotting. Moreover, immunofluorescence microscopy revealed lysosomal localization of Halo under DFO-induced ferritinophagic conditions, similar to endogenous FTH1 (Fig. 3, *D* and *E*). Significantly, colocalization observed in the control setting suggests that ferritinophagy is a process that is always occurring basally, as detected in Figure 1. In addition, we measured the generation of Halo fragment (TMR-HaloTag) after DFO treatment in a time-course setting. Our data show a time-dependent increase of HaloTag fragment after DFO treatment (Fig. 3, *F* and *G*). Taken together, these data indicate that Halo-FTH1 behaves similar to its endogenous counterpart, and detection of stabilized Halo fragment resulting from ferritinophagy can be used to detect the flux of this autophagic process. Importantly, depending on the expression levels of Halo-FTH1 in cells, Halo-FTH1 cleavage through ferritinophagy may be underappreciated because of limitations in the detection sensitivities. While we demonstrate the proof of principle, further optimization of expression level of Halo-FTH1 may be required based on cell lines and experimental setups in other laboratories.

**Figure 4:**
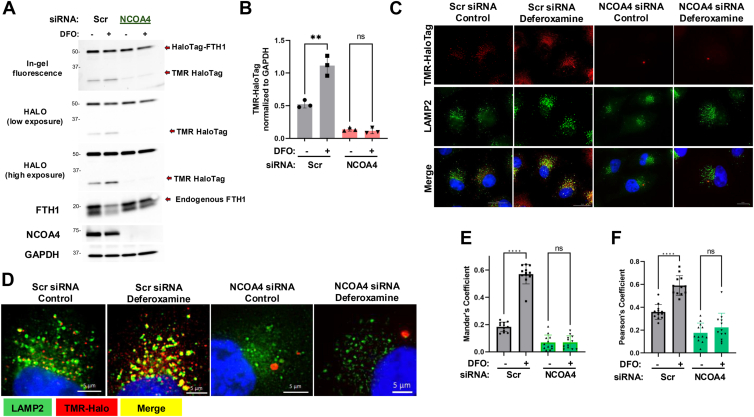
**Silencing of NCOA4 prevents ferritinophagy and Halo-FTH1 processing.***A* and *B*, Halo-FTH–expressing cells were transfected with control (Scramble) or NCOA4 siRNAs (5 nM, 24 h). Cells were treated with deferoxamine (DFO), and Halo-FTH1 and TMR-Halo complex, endogenous FTH1, and NCOA4 protein levels were detected using immunoblotting. TMR-Halo band intensities were quantitated and normalized to loading control (GAPDH). n = 3 independent experiments. Data represent mean ± SD. Statistical analysis was done by two-way ANOVA with Tukey’s multiple comparison tests. ns (nonsignificant) *p* > 0.05, ∗∗*p* < 0.05. *C* and *D*, cells were treated as in *A*, and colocalization of Halo fragment and lysosomes (LAMP2) was visualized using immunofluorescence microscopy. The scale bar represents 10 μm. *D*, high magnification images are presented. The scale bar represents 5 μm. *E* and *F*, colocalization of TMR–-alo with lysosomes in *C* and *D* are quantitated as Mander’s and Pearson’s coefficients. n = 12 in three independent experiments. Data represent mean ± SD. Statistical analysis was done by two-way ANOVA with Tukey’s multiple comparison tests. ns (nonsignificant) *p* > 0.05, ∗∗∗∗*p* < 0.05. FTH1, ferritin heavy chain 1; LAMP2, lysosome-associated membrane protein 2; NCOA4, nuclear receptor coactivator 4; TMR, tetramethylrhodamine.

NCOA4 acts as the adaptor protein in ferritinophagy, through its interaction with FTH1, delivering ferritin to lysosomes (14, 15). Therefore, we tested the role of NCOA4 in Halo-FTH1–expressing cells. Our data show that siRNA-mediated silencing of NCOA4 prevented DFO-induced accumulation of Halo fragment (TMR-HaloTag) and degradation of endogenous FTH1 (Fig. 4, *A* and *B*), consistent with its previously defined role (14, 15). In addition, lysosomal localization of Halo-FTH1 upon DFO treatment was also prevented after NCOA4 knockdown (Fig. 4, *C*–*F*). Importantly, NCOA4 silencing changed the cellular distribution of Halo-FTH1. While the number of Halo-FTH1 puncta significantly decreased, the size of these puncta appeared to be much bigger than the controls after NCOA4 silencing (Fig. 4*D*). It is possible that in the absence of NCOA4, ferritin cannot be transported to the lysosomes, and therefore, they form large condensates in the cytosol. Importantly, similar observations were made in cells lacking FIP200, ATG9A, or TAX1BP1, all of which participate in autophagy/ferritinophagy, where FTH1/ferritin formed large condensates in the cytosol (37, 38, 39). Moreover, fewer and enlarged puncta formation of FTH1/ferritin was also reported after silencing of NCOA4 (18, 38, 40), very similar to our observations. This change in the shape and number of ferritin puncta needs to be further evaluated in regulation of ferritinophagy and iron storage context. Nonetheless, our data strongly suggest that ectopically expressed Halo-FTH1 is a binding partner of NCOA4 that mediates its degradation in the lysosomes through ferritinophagy, same as the endogenous FTH1.

Altogether, the Halo-FTH1 tool we generated can be used to specifically detect and quantitate ferritinophagy in mammalian cells together with a fluorescent Halo ligand.

## Experimental procedures

### Cell lines and growth conditions

HeLa cervical cancer cells were provided by Dr Richard J. Youle (National Institute of Neurological Disorders and Stroke, National Institutes of Health [NIH]). Cells were grown in Dulbecco's modified Eagle's medium (Gibco; catalog no.: 11-965-092) with 10% fetal bovine serum (Bio-Techne; catalog no.: S12495) supplemented with sodium pyruvate (Corning; catalog no.: 25-000-CI) and GlutaMax (Gibco; catalog no.: 35-050-061) without antibiotics at 37 °C in a humidified incubator with 5% CO_2_. Possible mycoplasma contaminations were monitored regularly by MycoAlert kit (Lonza; catalog no.: LT07-318).

### Generation of pMRX-IP HaloTag7-FTH1

pMRX IP HaloTag7-LC3 (Addgene; #184899, a kind gift from Dr Noboru Mizushima) plasmid was digested with EcoRI (ThermoFisher Scientific; catalog no.: ER0271) and XhoI (ThermoFisher Scientific; catalog no.: ER0692) to obtain linearized plasmid backbone without LC3. FTH1 complementary DNA was amplified using the primers forward: TAACCTCGAGGAATTCCGGTACGACCGCGTC and reverse: TCGTGAATTCCTCGAGGTTAGCTTTCATTATCAC with the EcoRI and XhoI restriction sites. PCR product was ligated into the linearized pMRX IP HaloTag7 plasmid. The resulting plasmid pMRX IP HaloTag7 FTH1 was sequenced (Plasmidsaurus, Inc), ensuring lack of mutations.

### Generation of stable Halo-FTH1–expressing HeLa cells

Lentiviral particles encoding Halo-FTH1 were generated using pMRX-IP HaloTag7-FTH1, packing plasmids pCMV-VSV-G and pCMV-dR8.2 dvpr in human embryonic kidney 293FT cells. The resulting viral particles were used to transduce HeLa cells in the presence of 5 μg/ml polybrene for 48 h. Polyclonal stable cells were selected using 5 μg/ml puromycin.

### In-gel fluorescence and immunoblotting

Cells were collected in ice-cold 1X PBS and pelleted. Pellets were resuspended in cold lysis buffer (50 mM Tris–HCl [pH 7.5], 150 mM NaCl, 1 mM EDTA, 0.2% n-dodecyl-β-d-maltoside, protease and phosphatase inhibitors) and incubated on ice for 20 min before centrifugation at 12,000 rpm for 15 min at 4 °C. Protein concentrations were determined in the supernatants, and equal protein amounts were resolved by SDS-PAGE in 4% to 20% gradient PAGE gels (ThermoFisher; catalog no.: WXP42020BOX). The gels were washed three times with ddH_2_O, before visualizing for in-gel fluorescence using BIORAD ChemiDoc Imaging System (ALEXA 546 filter for 100 s). Proteins were transferred to polyvinylidene difluoride membranes, and immunoblotting was carried out as described previously (41). Cells were treated with 50 nM bafilomycin A1 overnight to inhibit lysosomal activity. The following antibodies were used: anti-FTH1 (Santa Cruz; catalog no.: SC-376594), anti-GAPDH (ProteinTech; catalog no.: 60004-1-Ig), Beta-Actin (ProteinTech; catalog no.: 66009-1-Ig), anti-HaloTag (Promega; catalog no.: G9211), and anti-NCOA4 (Cell Signaling; catalog no.: 66849). Blots were visualized using enhanced chemoluminescence using Azure 600 imager (Azure Biosystems). Band intensities were quantified and normalized to loading controls (GAPDH or actin) using ImageJ/FIJI (NIH).

### Immunofluorescence microscopy

HeLa cells stably expressing Halo-FTH1 (30,000 cells) were seeded into 35 mm dishes, on poly-d-lysine-coated glass coverslips. The next day, cells were treated with DFO (Sigma–Aldrich; catalog no.: D9533) (50 μM) for 16 h. After the treatments, the cells were labeled with 100 nM HaloTag TMR ligand (Promega; catalog no.: G8252) for 2 h in dark before fixation with 4% paraformaldehyde in 1X PBS at room temperature for 10 min. Immunofluorescence confocal imaging was carried out as described (41, 42) using anti-FTL antibody (ProteinTech; catalog no.: 10727-1-AP), anti–lysosome-associated membrane protein (Santa Cruz; catalog no.: sc-18822). Nuclei were stained using 4',6-diamidino-2-phenylindole (Abcam; catalog no.: ab104139), anti-Rabbit IgG Alexa Fluor 647 (ThermoFisher; catalog no.: A31573), and anti-Mouse IgG Alexa Fluor 488 (ThermoFisher; catalog no.: A21202). Slides were visualized using KEYENCE (BZ-X700) fluorescence microscope. Images were quantified in ImageJ (NIH) using JacoP plugin (43).

### FerroOrange staining and imaging

Cells seeded into poly-d-lysine–coated glass coverslips were treated with DFO. Following treatments, cells were washed with Hank’s balanced salt solution and labeled with 1 μmol/l of FerroOrange (Dojindo; catalog no.: F374-10) in serum-free media at 37 °C incubator for 30 min. After Hank’s balanced salt solution washes, slides were visualized using KEYENCE (BZ-X700) fluorescence microscope. Images were quantified using ImageJ (NIH), which measured raw intensity density normalized to the area.

### siRNA transfections

Nontargeting control (Scrambled) siRNA (Qiagen; catalog no.: 1027281) or SMARTpool siRNA targeting NCOA4 (Dharmacon; catalog no.: M-010321-01-0005) were transfected into the cells at 5 nM final concentration using Lipofectamine RNAiMAX (ThermoFisher; catalog no.: 13778100) as described by the manufacturer.

### Statistical analyses

All data are presented as means ± SD of at least three independent studies (n ≥ 3). Group comparisons were performed with two-tailed unpaired *t* tests for comparison of two groups and with two-way ANOVA for comparison of more than two groups with Tukey’s multiple comparison tests using GraphPad Prism software (Dotmatics), version 9.4.1. *p* < 0.05 was considered significant.

## Data availability

All the data are contained within the article.

## Conflict of interest

The authors declare that they have no conflicts of interest with the contents of this article.
